# Establishing a Preparedness Program: Lessons From COVID-19 to Strengthen Global Strategies for Future Pandemics

**DOI:** 10.7759/cureus.97402

**Published:** 2025-11-21

**Authors:** Ata Mohajer-Bastami, Sarah Moin, Suhaib Ahmad, Ahmed Ahmed, Anurag Agarwal, Sjaak Pouwels, Mohammed Hammoda, Wah Yang, Aristomenis K Exadaktylos

**Affiliations:** 1 Surgery, Ealing Hospital, London, GBR; 2 General Surgery, East Surrey Hospital, Redhill, GBR; 3 General Surgery, Health Education and Improvement Wales, Bangor, GBR; 4 General Surgery, Imperial College London, London, GBR; 5 General Surgery, Apollo Hospitals, Bangalore, IND; 6 General Surgery, Betsi Cadwaladr University Health Board, Bangor, GBR; 7 Intensive Care Medicine, Elisabeth-Tweesteden Hospital, Tilburg, NLD; 8 General Surgery, Health Education and Improvement Wales, Swansea, GBR; 9 Surgery, The First Affiliated Hospital of Jinan University, Guangzhou, CHN; 10 Emergency Medicine, University of Cyprus, Nicosia, CYP

**Keywords:** covid-19, epidemiological models, global health security, pandemic preparedness, public health response, sars-cov-2 transmission, surveillance systems, vaccine equity

## Abstract

This study aimed to evaluate the global response to the COVID-19 pandemic, identify preparedness gaps, and propose strategies to strengthen resilience for future health crises, drawing on the natural history of SARS-CoV-2 and epidemiological models. The research team conducted a narrative review with a descriptive analysis supported by case examples and modeling. We synthesized published literature, official reports, and surveillance data, analyzing COVID-19 transmission using the epidemiological triangle model. Comparative case studies from South Korea, New Zealand, and the United Kingdom were examined to assess intervention effectiveness. National surveillance systems, outbreak response mechanisms, and public communication strategies were critically reviewed.

Our findings show that disparities in testing, data transparency, and infrastructure shaped outcomes globally. Surprisingly, many high-income countries underperformed despite prior preparedness rankings. Countries implementing early lockdowns, equitable vaccine rollouts, and consistent public messaging achieved better control. However, asymptomatic transmission, weak surveillance integration, and delayed policy actions hindered containment. Vaccine inequity and fragmented data systems further limited coordinated response. Future preparedness must emphasize early interventions, sustained surveillance investment, transparent communication, and equitable access to care. Lessons from COVID-19 underscore the value of multidisciplinary response teams, international data sharing, and targeted testing strategies to mitigate the impact of future pandemics.

## Introduction and background

The COVID-19 pandemic has provided invaluable lessons that can strengthen global preparedness for future public health emergencies. While no single country had a perfect strategy, collective experiences have highlighted the critical elements of surveillance, rapid response, and outbreak management. It has had a profound impact on global health, economies, and societal structures, highlighting the critical need for comprehensive preparedness, readiness, and control measures. The rapid spread of the virus resulted in millions of infections and fatalities, overwhelming healthcare systems and straining medical resources. Economically, the implementation of lockdowns and movement restrictions led to profound disruptions in global trade, labor markets, and financial stability, further exacerbating existing socio-economic disparities. The pandemic also had far-reaching social implications, including heightened mental health concerns, interruptions in education, and disparities in access to essential healthcare services, disproportionately affecting vulnerable populations [1]. These challenges underscore the necessity for enhanced international collaboration, resilient public health systems, and adaptive policy frameworks to mitigate the impact of future pandemics and strengthen global health security.

Despite our advances in the biomedical field and vaccine development, the pandemic greatly exposed existing structural weaknesses in global health governance. The World Health Organization’s (WHO) 2023 Preparedness and Resilience for Emerging Threats (PRET) framework and the Global Preparedness Monitoring Board emphasized that health security cannot depend solely on emergency response capacity but rather must be integrated into long-term planning of strengthening current systems [2,3]. Governments must transition from reactive strategies to proactive policies, incorporating evidence-based recommendations into national and international preparedness frameworks. The Lancet COVID-19 Commission concluded, similarly, that the crisis was "a massive global failure of policy coordination and social solidarity," demonstrating that a good preparedness program is not reliant on scientific advancement alone but requires an additional balance of political will, public trust, and engagement.

This study aimed to discuss pandemic preparedness and response, addressing the natural history of COVID-19 through the use of epidemiological models. Additionally, it will analyze the global impact of COVID-19, encompassing both health and socio-economic consequences. This paper will also offer recommendations for future public health strategies to enhance global resilience against pandemics. By synthesizing lessons learned from COVID-19, these recommendations will aim to strengthen pandemic preparedness and response efforts, ensuring a more effective and coordinated global approach to managing future public health threats. This narrative review synthesizes descriptive epidemiological trends and healthcare system response.

Although this is a narrative review, we followed a structured approach to identify relevant literature. A targeted search was conducted using Medline and Embase databases. Keywords included "COVID-19," "pandemic preparedness," "public health response," "lockdown strategies," and "surveillance systems." Inclusion was based on relevance to the pandemic response or preparedness strategies. Only peer-reviewed studies were included in this narrative review.

Natural history of COVID-19

COVID-19 is caused by the currently named SARS-CoV-2 virus, belonging to the coronavirus family [4]. There has been evidence that it is spread through aerosol, fecal-oral contact, and through fomites. However, the predominant method of spreading the disease has been respiratory droplets from person to person [5].

To enhance the understanding of COVID-19 transmission and control measures, this study will utilize the epidemiological triangle model. This model effectively illustrates the fundamental principles of disease spread by identifying the following three critical components: the agent (SARS-CoV-2), the host (human populations), and the environment (conditions facilitating transmission). The use of the epidemiological triangle allows for a clearer comprehension of how targeted public health measures, such as vaccination, social distancing, and improved hygiene practices, can mitigate disease spread. This analysis will serve as a foundation for discussing key interventions aimed at reducing COVID-19 transmission in subsequent sections of this study.

Around 80% of infected cases of COVID-19 show no or mild flu-like symptoms and have a full recovery [4]. Following an incubation period of 10-14 days, some infected individuals can move on to the "pulmonary disease" phase, where the patient experiences chest symptoms, such as a continuous cough or shortness of breath, and can be seen radiologically on X-ray or CT scans [6]. The subsequent stage of disease is the "extra" or "hyper pulmonary phase," whereby the virus can affect other vital body organs beyond the lungs.

## Review

Impact of COVID-19

All humans are susceptible to COVID-19. Individuals who are elderly, male sex, and/or have any underlying chronic health conditions, particularly respiratory or cardiovascular disease, are at a higher risk of developing complications or requiring hospitalization [7]. The expanding elderly population and the associated rise in mortality rates among high-risk older individuals highlight a critical challenge. By 2030, it is estimated that 21.8% of the United Kingdom population will be aged 65 years or over, and 6.8% will be aged 75 years or over [8,9].

The global response to the COVID-19 pandemic saw widespread lockdowns across numerous countries, with China being the first to implement such measures in Wuhan on January 23, 2020, as the virus began to spread. The spread of the virus was further facilitated by documented evidence highlighting that China had not admitted to the leak in sufficient time, and there was a degree of censorship that, allegedly, citizens needed to circumvent in order to get further information, with the delay causing negative effects [10].

By March 2020, several European countries, including Italy and Spain, had strict national lockdowns to mitigate the virus's transmission, and many other nations imposed similar restrictions in the subsequent weeks [11]. According to the United Nations, by April 2020, nearly 90% of the world’s population was living under some form of lockdown or social distancing measures, highlighting the global scale of the response [12].

Certain countries implemented mass testing and digital contact tracing early on during the pandemic. Measures included drive-through testing centers, mask mandates, and clear transparent communication. Furthermore, countries like New Zealand introduced strict lockdowns at an early stage and adopted an alert system, supported by strong political leadership and border closures. In both cases, early decisions were the key to success [11].

The impact of the pandemic was unprecedented, affecting not only mortality rates but also various sectors worldwide. In the healthcare sector, the surge in COVID-19 cases led to an extraordinary increase in demand for hospital beds, with each subsequent wave of infections straining healthcare systems globally. There has been an indirect effect on the NHS from this; according to the British Medical Association (BMA), the number of patients waiting more than 12 hours for hospital admission in March 2022 was 10 times higher than it was in March 2020. There are currently around seven million people on the NHS waiting list for consultant-led specialist care; almost double the 4.43 million who were on the same list in February 2020 [13].

Beyond health and healthcare, COVID-19 has shown a series of social, economic, and cultural effects, which will each have their own impact. The pandemic has shown evidence of exposing and further worsening social inequalities. An extensive report looking into this found that individuals from certain areas and certain communities have been made even more vulnerable and disadvantaged than they were previously [14,15]. The impact on health and well-being, cohesion within communities, and the economy will likely have affected the world for years to come.

The psychological burden of the pandemic was also considerably substantial with the World Health Organization (WHO) reporting a 25% global increase in the prevalence of anxiety and depression [16]. Prolonged social isolation and loss of employment contributed to a "mental health pandemic," which disproportionately affected younger adults, healthcare workers, and socially disadvantaged groups, further widening health disparities.

Given the devastating impact of COVID-19 worldwide, it is crucial to ensure adequate preparedness for future outbreaks. This highlights the need for a comprehensive and effective preparedness program that can mitigate the effects of such global health crises. In the following section, we will discuss the key components and strategies that should constitute an optimal preparedness framework.

Description of optimal preparedness program

A good understanding of planning and surveillance, along with a clear set of guidance for an ideal preparedness program, would help limit the profound impact a disease could have on a country. A good program would include setting action goals simultaneously with an essential surveillance program. Surveillance is a system that is run by public health organizations in collaboration with other professionals. It is defined as a "continuous, systematic collection, analysis, and interpretation of health-related data" [17]. The key components of a surveillance system are that it is active and continuous, and most importantly, it provides essential information to guide further action. The data collected by public health agencies can demonstrate early warning signs of an impending public health emergency and analyze the impact of an intervention. The key aspect is to ensure that surveillance is ongoing, thus providing more details of the epidemiology of the disease, which can further guide strategies for further intervention. This information can then be disseminated to the relevant healthcare systems for action to take place.

The importance of a surveillance system is further demonstrated by the "iceberg phenomenon." The model is used to explain that reported cases are only the "top of the iceberg," where in reality there may be many more positive cases, which remain undiagnosed in the community [18]. These cases are likely to be undiagnosed as they may have milder symptoms and therefore are less likely to seek clinical attention. Surveillance systems allow for the "submerged" portion of the iceberg, which includes infected individuals who have not reported a positive case to be counted.

A good surveillance reporting system would rely on laboratory reporting as well as clinician reporting. Clinicians can report notifiable diseases to the relevant authority if there is clinical suspicion of the disease based on signs and symptoms. Laboratories can also notify positive samples that are found through testing. Surveillance information can then be analyzed by time, place, and person. This information can then be analyzed and disseminated to policymakers in a timely manner so that effective intervention programs can be implemented swiftly [19].

Alongside surveillance to provide ongoing analysis of relevant information to feedback to relevant actions, there should ideally be steps in place to control an outbreak of a disease. Public Health England produced an operational guidance framework for communicable disease outbreak management [20]. The steps in outbreak control would include confirming an outbreak and verifying the disease in question. In the context of COVID-19, a novel virus in the United Kingdom, an outbreak would be two or more epidemiologically linked cases of COVID-19. The next steps would involve setting up an outbreak control team with relevant professionals and forming a wider multidisciplinary approach to controlling the outbreak. The specifics of the outbreak control team will be addressed in greater detail later. Active case finding should then be implemented. As discussed, not all cases will always be visible to surveillance, as some clinicians may fail to or not know that they should report cases, not every individual will seek medical attention, and laboratories may issue false-negative test reports. Active case finding should include additional sources of data, including hospital admission date, school attendance registers, and workplace human resource department reports.

With global pandemic diseases, such as COVID-19, one should consider utilizing case definitions published by global health agencies such as the WHO. The WHO defined COVID-19 cases based on both positive antigen test results and based on clinical symptoms in combination with epidemiological criteria [21]. The issues with COVID-19 are that its symptoms closely resemble those of other milder viral respiratory conditions. The relevance of adding the epidemiological case definition by the WHO (that is, working or residing in an area with transmission and/or an area with high risk of transmission of COVID-19) is so important. It has been found that asymptomatic carriers can be as infectious as symptomatic cases [22]. To overcome this, alongside good surveillance should be high rates of testing for both symptomatic and asymptomatic individuals who are at high risk due to recent contact or working in healthcare settings. Although the risk of hospitalization and death was lower for younger people, there is a clear necessity to reduce their role in community transmission.

The remaining steps would be to develop and test hypotheses in the form of research. Defining the outbreak using descriptive epidemiology, which involves describing cases with regard to time, place, and person, can both help generate a testable hypothesis in the form of a treatment or immunization and measure the progress of the control program [23]. The generation of vaccinations as well as treatments is essential for primary, secondary, and tertiary prevention methods in the public. This will be further explored in the discussion of the recommended policies.

As previously mentioned, the outbreak control team would be responsible for leading the outbreak response. Typically, the members would include a chair, an epidemiologist, a microbiologist, environmental health workers, and communication teams. Each has a critical role to play. The communication team members coordinate messages from the outbreak control team to relevant bodies. They work closely with the media and can help influence people’s behaviors to help limit the spread. A review found the importance of media, particularly social media, in engaging with the public and helping quash the spread of "fake news" with false information on COVID-19 [24]. Changing behavior is a difficult task. With the aid of frequent presentation of up-to-date epidemiological data and spreading messages of recommendations, such as social distancing to prevent the spread of the disease via droplets, can be widespread via media outlets. A report in the United States has highlighted the role of the environmental health workforce as part of the multidisciplinary team and how they were essential in the COVID-19 pandemic [25]. The education of healthcare staff is important in health crises, as highlighted in assessments of frontline nursing knowledge in infectious disease control [26]. In global pandemics, it is essential to seek guidance from organizations such as the WHO while benchmarking relevant information against both domestic and international data. This approach facilitates the sharing and utilization of critical data, enhancing the overall response by incorporating insights from various countries.

The pandemic has demonstrated the importance of economic safeguards. We can easily notice how fragile the global supply chains can be, especially for medical goods. The focus should shift to creating contingency plans, such as emergency funding reserves, small-business support, and continuity of critical services. Furthermore, it is important to diversify supply chains and have a local manufacturing capacity for PPE and pharmaceuticals.

Critical review of the pandemic preparedness program

The United Nations and the WHO define preparedness as "the ability (knowledge, capacities, and organizational systems) of governments, professional response organizations, communities, and individuals to anticipate, detect, and respond effectively to, and recover from, the impact of likely, imminent, or current health emergencies." Effective pandemic response mechanisms should address key components, including detection, assessment, treatment, escalation, and recovery. These strategies must be continuously evaluated and adapted to reflect the most recent scientific advancements and epidemiological trends [27].

The 2019 Global Health Security Index evaluated pandemic preparedness across nations and ranked several high-income countries among the best prepared [28]. However, despite this recognition, the COVID-19 pandemic exposed significant gaps in preparedness across multiple regions. Multiple reviews found that despite high Global Health Security Initiative (GHSI) scores, many high-income nations failed in providing an adequate preparedness program due to factors such as fragmented government infrastructures, inconsistent public messages and updates, and diminished trust between the public and higher authorities [29,30]. Conversely, lower-income countries maintained relatively lower mortality rates from rapid mobilization and stronger community trust and engagement, suggesting political coordination and social cohesion were stronger predictors of success than GDP.

Countries did not account for public trust, misinformation resilience, or healthcare workforce flexibility, all of which are crucial. For instance, the United Kingdom (UK) conducted a cross-governmental pandemic simulation, Exercise Cygnus, in October 2016. The exercise revealed major vulnerabilities, including shortages in intensive care unit (ICU) beds and personal protective equipment (PPE), as well as a lack of coordinated response plans at local and national levels [31]. Despite these findings, few substantive changes were implemented before the emergence of COVID-19, reflecting a common issue worldwide where preparedness assessments are not always translated into actionable policies. Future preparedness should integrate real-world simulations, testing responsiveness under uncertainty, and include social equity and improved communication.

One of the most critical elements in the global response to COVID-19 was the rapid development and deployment of vaccines. The approval of emergency-use vaccines, beginning with the UK’s authorization in December 2020, marked a significant achievement in pandemic response [32]. However, disparities in vaccine distribution and uptake emerged as a global challenge [33]. Studies indicated that marginalized communities, including minority ethnic groups in high-income countries, had lower vaccination rates despite being disproportionately affected by COVID-19 [34]. Vaccine inequity remains one of the pandemic’s lasting failures. By the middle of 2021, high-income countries had secured over 70% of available vaccine doses globally, while low-income countries had not vaccinated even 10% of their populations [35]. Although there were existing initiatives aimed at distributing doses equally, success was limited due to global supply chain barriers and export restrictions.

An essential part of both surveillance and active case finding is community testing. The WHO emphasized the importance of widespread testing to identify asymptomatic cases and curb community transmission. However, several nations faced challenges in scaling up testing capacity in the early stages of the pandemic. In contrast, countries like South Korea and Australia rapidly implemented mass testing programs, contact tracing, and digital tracking mechanisms, leading to more effective containment [36]. These disparities underscore the importance of pre-established laboratory networks, supply chains, and flexible policy frameworks that allow for rapid scale-up of testing capacity in response to emerging threats.

Various approaches to testing had measurable effects. Countries including Italy and Spain, which had a delayed lockdown, witnessed significantly higher early death rates compared to places like New Zealand and Taiwan, which acted quickly. Some countries, such as South Korea, managed to avoid a full national lockdown by expanding testing and tracing early. On the other hand, Germany demonstrated consistent national messaging, improving public trust, while more fragmented approaches, such as ones in the United States and United Kingdom, led to greater public confusion and resistance [11].

Recommendations for public health strategy and policy against COVID-19

The COVID-19 pandemic has demonstrated the necessity of a robust and adaptive public health response on a global scale. Given the unpredictability of future waves of infection, countries must be prepared for worst-case scenarios through evidence-based strategies. The following recommendations focus on key areas of intervention, including timely implementation of control measures, ongoing research, equitable vaccine distribution, effective communication, and sustained surveillance and testing.

Rapid implementation of control measures

The prompt introduction of control measures is essential in mitigating the spread of COVID-19. Some nations, such as New Zealand and South Korea, implemented early and strict lockdowns, which significantly reduced transmission rates [37]. Conversely, the UK’s initial response was delayed compared to other nations, contributing to the increased severity and duration of the first wave [38]. Future responses must prioritize the rapid implementation of restrictions based on real-time epidemiological data to minimize the impact of subsequent waves worldwide.

Simulation modeling demonstrated that for every one-week delay in lockdown implementation, there was an associated 30% increase in mortality in European countries [39]. This study argues that any future policies must incorporate adaptive decisions informed by real-time epidemiological data rather than political timelines.

Continuous research and scientific advancements

Ongoing research into the virology, transmission dynamics, and clinical manifestations of SARS-CoV-2 remains critical. The rapid development of vaccines within a year of the virus’s emergence underscores the importance of scientific inquiry. Continued global investment in research will facilitate the advancement of therapeutics, vaccines, and disease interventions, thereby enhancing preparedness for future variants and emerging infectious diseases across all nations. Recent studies have highlighted the role of artificial intelligence in pandemic response efforts, such as early detection, prediction, and optimizing healthcare resources [40].

Addressing disparities in vaccine uptake

Discrepancies in vaccine uptake are a global concern, particularly among marginalized and low-income communities. Vaccine hesitancy is a major barrier, with a recent meta-analysis identifying exposure to misinformation and political polarization as primary drivers [41]. Effective strategies may include co-creation of messages with community leaders and transparent communication of vaccine safety data to build public trust.

In the United Kingdom, for example, vaccine hesitancy was notably higher among Black, Asian, and Minority Ethnic (BAME) communities [42]. Addressing vaccine hesitancy through community engagement, public awareness campaigns, and targeted immunization efforts is critical. Until widespread vaccination is achieved, priority should be given to high-risk groups, including the elderly, individuals with chronic health conditions, and underserved populations worldwide. Limited supplies of emerging treatments should similarly be allocated based on clinical need to maximize their impact.

Effective public communication strategies

Transparent and consistent communication is fundamental to public compliance with health measures. The spread of misinformation or "infodemics" poses a significant challenge to pandemic management globally [24]. Public messaging should leverage mainstream media and social media platforms to disseminate clear, evidence-based information [42]. Additionally, public health guidance should be simplified and revised only as necessary to minimize confusion and frustration, which have previously undermined compliance [43]. Countries should adopt strategies that consider cultural and linguistic diversity to ensure effective communication with all populations.

Sustained surveillance and testing

Long-term surveillance and widespread testing are essential for detecting new waves of infection and identifying at-risk populations. Epidemiological monitoring provides critical data for targeted interventions, including localized restrictions and resource allocation. Community testing should continue, particularly for asymptomatic carriers, to prevent silent transmission. Weekly testing of healthcare and care home staff, as well as residents, should be implemented in all high-risk settings to prevent outbreaks and enable timely isolation and treatment of positive cases. Global cooperation in genomic surveillance is also crucial in identifying and responding to new variants.

The recommendations outlined above emphasize the importance of swift intervention, continued research, equitable vaccine distribution, effective communication, and rigorous surveillance. By implementing these strategies, countries worldwide can strengthen their preparedness and resilience against future waves of COVID-19 and other emerging infectious diseases.

## Conclusions

Scientific research had long predicted the likelihood of a pandemic arising from zoonotic transmission, as evidenced by a 2008 Home Office report. However, despite early warnings, responses often deviated from established guidelines set by national authorities and the WHO. Strengthening healthcare infrastructure, ensuring equitable vaccine distribution, and investing in real-time surveillance systems are essential components of an effective response. Furthermore, international collaboration and transparent information-sharing mechanisms will play a crucial role in mitigating the impact of future pandemics.

Looking ahead, the establishment of a comprehensive preparedness program is crucial. By addressing these systemic gaps, the global community can enhance resilience against emerging infectious disease threats and improve overall public health security. The experience of COVID-19 underscores that pandemic preparedness is not merely a technical challenge but a societal one, requiring cooperation across political, economic, and cultural boundaries. Investing in global health equity, digital interoperability, and public trust will determine the world’s resilience against the next inevitable pandemic.
